# Benefits of Cohort Studies in a Consortia-Dominated Landscape

**DOI:** 10.3389/fgene.2021.801653

**Published:** 2021-12-07

**Authors:** Selam Zenebe-Gete, Rebecca Salowe, Joan M. O’Brien

**Affiliations:** Department of Ophthalmology, Scheie Eye Institute, University of Pennsylvania, Philadelphia, PA, United States

**Keywords:** GWAS - genome-wide association study, consortia, cohort, study design, genetics, big data

## Introduction

Since their inception in 2005, Genome-Wide Association Studies (GWAS) have risen in popularity as a key method for gene discovery. Efforts to make use of existing genetic data, identify the causes of complex disease, and improve study power by increasing sample sizes have led to the formation of many GWAS consortia (Lutz et al., 2013). Consortia can bypass major logistical and financial challenges that accompany the recruitment of large study populations (Benjamin et al., 2018). Additionally, data sharing via consortia can help researchers diversify their study populations and can encourage research collaborations. While consortia do circumvent several financial, logistical, statistical, and demographic challenges to conducting GWAS, the collective swing towards their formation leaves several gaps in the field of genetics.

The majority of GWAS, including both small cohort studies and large consortia, often fail to identify actionable genetic determinants of complex disease. Only 2.2% of GWAS conducted between 2005 and 2016 had follow-up functional studies (Gallagher and Chen-Plotkin, 2018). Additionally, over 90% of phenotype-associated single nucleotide polymorphisms (SNPs) discovered in GWAS are in non-coding regions of the genome (Qu and Fang, 2013), with their effects on clinical outcomes either unknown or under investigation. Additionally, of the variants identified as statistically significant, a subset may not be clinically actionable because of their essential roles in cellular function. For example, p53 plays a significant role in many disease pathways (i.e. cancer), but its regulatory functions in all cells makes it a poor target for drug therapy.

Though these limitations apply to most GWAS, there are several unique advantages to cohort studies, including: 1) discovery of clinically actionable targets through subtyping; 2) the necessity of post-GWAS follow-up on variants; and 3) identification of population-specific findings.

## Subsections

### Clinically Actionable Variants

Stratifying study populations by disease subtype may facilitate identification of clinically actionable variants. The discovery of variants through subtype-stratified GWAS has been well documented in the literature. In breast cancer research, stratified GWAS have identified subtype-defining SNPs, such as variants in the *HER2* gene (O’Brien et al., 2014). The development of therapies targeting HER2 pathways improved outcomes for this subtype of breast cancer (Arteaga et al., 2012; Figueroa-Magalhães et al., 2014). Similarly, in an ischemic stroke study, all statistically significant variants were subtype-specific (Traylor et al., 2012). Subtype-stratified GWAS of bipolar disorder (BD) (Charney et al., 2017), autism spectrum disorder (ASD) (Hu et al., 2011), and Parkinson’s disease (von Coelln and Shulman, 2016) patients also led to identification of novel disease subtype SNPs.

While subtyping can be implemented in both cohort and consortia studies, cohort studies may more easily accommodate subtyping methods, particularly when investigating complex diseases. Since some complex diseases do not have clearly defined subtypes, individual studies may define subtypes using different criteria. For example, a lack of methodological standardization in neuroimaging of Alzheimer’s patients has hindered efforts towards consistent subtyping (Mohanty et al., 2020). Data harmonization methods are then needed to address differences in subtype classification between study cohorts within a consortium, increasing risk of introducing bias. A cohort study avoids this potential source of bias since it operates under a single set of subtyping criteria. Additionally, many cohort studies investigate a single disease type (i.e., open-angle glaucoma) rather than larger combinations (i.e., all forms of glaucoma), allowing for further subtyping of one form of disease. Since different forms of this disease may have different biological causes, putting them together to increase power may yield results that do not have biologic or physiologic relevance. Well-defined subtype-stratified GWAS in cohort studies may improve researchers’ attempts to identify clinically actionable disease targets compared to consortia-based GWAS.

### Post-Genome-Wide Association Study Follow-Up on Variants

Cohort studies tend to be more amenable to collecting longitudinal data and conducting follow-up on variants of interest (Wijmenga and Zhernakova, 2018). As more variants have been identified by GWAS, interest in investigating them further through functional studies, “multi-omic” research, and other “post-GWAS” methods has peaked. Cohort studies’ focus on a single, typically local study population allows researchers to re-contact patients with interesting variants for follow-up. Cohort studies also allow for collection of longitudinal phenotypic data, which is key to the study of disease progression. Since consortia patients originally enrolled in studies at a variety of sites, patient re-contact and collection of longitudinal data are complicated and may not be possible in studies of such a wide scale. Furthermore, information collected at each site may lack consistency in data type and collection method, requiring additional harmonization efforts to make them comparable. For example, individual studies within the Psychiatric GWAS Consortium used different genotyping platforms, requiring imputation against existing gene expression data to standardize their genotypes (Sullivan, 2010). Even basic phenotypic parameters such as age and diet can affect an individual’s gene expression (Wijmenga and Zhernakova, 2018), highlighting another difference between populations that can complicate combining multiple populations in consortia.

### Population-Specific Variants

Cohort studies can also aid in identifying variants that are specific to minority populations, who remain dramatically under-represented in genetic studies. Population-specific GWAS are important to both understanding the genetics of complex traits and to elucidating the role of specific variants in minority populations (Sirugo et al., 2019). Studies have shown that differences in ancestry contribute to variations in disease prevalence, severity, and resistance across populations (Haga, 2010). However, while extensive GWAS testing has been done among individuals of European-descent, investigations of similar scale have not been conducted in African ancestry populations (Campbell and Tishkoff, 2008), with only 3% of GWAS participants being of African descent as of 2016 (Popejoy and Fullerton, 2016).

Although some consortia can increase study population diversity through collaborative efforts to catalog human genetic variation, such as the 1000 Genomes Project (Jankovic et al., 2010), extensive data compilation may prevent identification of variants that are specific to underrepresented groups when they are un-stratified within the larger population. For example, an investigation of 3,899 SNPs in 313 genes in self-identified Caucasians, African Americans, Asians, and Hispanics found distinct and non-overlapping clustering of the Caucasian, African American and Asian samples (Stephens et al., 2001). This finding suggests differing genetic architecture between these groups, supporting the need for ancestry-specific genetic studies. Additionally, recruiting patients from a single city, as many cohorts do, minimizes the differences within a demographic group. Self-identified African Americans differ in their genetic admixture across different geographic locations (Bryc et al., 2015), suggesting that single city studies have less population heterogeneity than consortia.

## Discussion

While consortia do play an important role in genetic research, cohort-based studies may be better suited to identifying clinically actionable disease pathways and studying underrepresented minority populations. Although the recent publication climate emphasizes large consortia, GWAS of specified cohorts may produce more precise results that can be used in studies aiming to link genetics to endophenotypic data over time. Returning to the example of breast cancer, underlying pathology and genetics are now used to subtype patients in order to personalize treatment. One consortium including over 120,000 breast cancer patients identified 65 novel loci associated with overall breast cancer risk via GWAS (Michailidou et al., 2017). However, advancements in breast cancer technology have demonstrated the importance of molecular subtyping in patient prognosis and treatment (Yang and Polley, 2019). When multiple diseases or subtypes with distinct molecular pathways are categorized together in GWAS, as in the above study, the resulting genetic findings may not be clinically actionable. Putting this into context, future genetic studies may benefit from refocusing on the end goal of all GWAS—not only to find statistical significance, but to identify variants with the potential to improve health outcomes. Improving the accuracy of GWAS findings and translating these results to the clinic may be facilitated through a greater balance between both consortia- and cohort-based methods.
